# Outcome Uncertainty and Brain Activity Aberrance in the Insula and Anterior Cingulate Cortex Are Associated with Dysfunctional Impulsivity in Borderline Personality Disorder

**DOI:** 10.3389/fnhum.2016.00207

**Published:** 2016-05-06

**Authors:** Jørgen Assar Mortensen, Hallvard Røe Evensmoen, Gunilla Klensmeden, Asta Kristine Håberg

**Affiliations:** ^1^Department of Neuroscience, Norwegian University of Science and Technology (NTNU)Trondheim, Norway; ^2^Tiller Psychiatric Center, St. Olavs HospitalTrondheim, Norway; ^3^Department of Research, NordlandssykehusetBodø, Norway; ^4^Department of Medical Imaging, St. Olavs HospitalTrondheim, Norway

**Keywords:** intolerance for uncertainty, disinhibition, behavioral dysregulation, neuronal correlates, mental disorders

## Abstract

Uncertainty is recognized as an important component in distress, which may elicit impulsive behavior in patients with borderline personality disorder (BPD). These patients are known to be both impulsive and distress intolerant. The present study explored the connection between outcome uncertainty and impulsivity in BPD. The prediction was that cue primes, which provide incomplete information of subsequent target stimuli, led BPD patients to overrate the predictive value of these cues in order to reduce distress related to outcome uncertainty. This would yield dysfunctional impulsive behavior detected as commission errors to incorrectly primed targets. We hypothesized that dysfunctional impulsivity would be accompanied by aberrant brain activity in the right insula and anterior cingulate cortex (ACC), previously described to be involved in uncertainty processing, attention-/cognitive control and BPD pathology. 14 female BPD patients and 14 healthy matched controls (HCs) for comparison completed a Posner task during fMRI at 3T. The task was modified to limit the effect of spatial orientation and enhance the effect of conscious expectations. Brain activity was monitored in the priming phase where the effects of cue primes and neutral primes were compared. As predicted, the BPD group made significantly more commission errors to incorrectly primed targets than HCs. Also, the patients had faster reaction times to correctly primed targets relative to targets preceded by neutral primes. The BPD group had decreased activity in the right mid insula and increased activity in bilateral dorsal ACC during cue primes. The results indicate that strong expectations induced by cue primes led to reduced uncertainty, increased response readiness, and ultimately, dysfunctional impulsivity in BPD patients. We suggest that outcome uncertainty may be an important component in distress related impulsivity in BPD.

## Introduction

Borderline personality disorder (BPD) is characterized by impulsive actions particularly under distress (Tragesser and Robinson, 2009; Sebastian et al., 2013), as well as emotional dysregulation (American Psychiatric Association, 2013). Persons with BPD are known to have low distress tolerance (Linehan, 1993), which may lead to dichotomous thinking, as clinically manifested in either idealization or devaluation (Kernberg, 1975; Linehan, 1993; Veen and Arntz, 2000). According to Kernberg (1975), dichotomous thinking (or splitting) entails a minimization of uncertainty related to the internal representations of the world in order to make them less distressful. Meanwhile, this may give rise to strong certainty and beliefs, and a narrowing of attention to features of specific relevance for these beliefs (Avila, 1995; Yu and Dayan, 2005; Feldman and Friston, 2010). Subsequently, it may lead to rash, impulsive actions (Avila and Parcet, 2002). The connection between uncertainty, attention and impulsivity is yet unexplored in the neurobiological study of BPD.

The previously revealed BPD related activity aberrance in limbic and prefrontal decision making networks, may shed light on this connection (Krause-Utz et al., 2014). In particular, the anterior part of insula, the adjoining operculum, the middle/superior frontal gyri, and the anterior cingulate cortex (ACC) contain the unique von Economo neurons, whose large axons facilitate rapid relay and thus provide fast control signals to other cortical regions (Allman et al., 2005; Dosenbach et al., 2008; Fajardo et al., 2008; Menon and Uddin, 2010). The posterior part of insula monitor visceral, somatosensory and motor information and informs the mid-anterior part, including the operculum, which integrates these signals into conscious feeling states (Damasio, 1996; Craig, 2009). Insula is proposed to use the interoceptive information to engage externally oriented attention and internal cognitive control, sustained by ACC and the middle/superior frontal gyri, in order to facilitate an appropriate behavioral response (Menon and Uddin, 2010). While the left insula is associated with parasympathetic activity and positive affect, the right side is associated with sympathetic activity and distress (Craig, 2005). In addition, the right mid-anterior side, specifically, is demonstrated to become activated under conditions of uncertainty (Paulus and Frank, 2006; Simmons et al., 2008; Sarinopoulos et al., 2010; Bach and Dolan, 2012; Becker et al., 2014) and has previously been related to distress in BPD patients (Dziobek et al., 2011) as well as in patients with anxiety disorders (Etkin and Wager, 2007). Moreover, a right mid-anterior activity increase has been associated with difficulties in response inhibition in both BPD patients (Silbersweig et al., 2007) and non-BPD subjects (Dambacher et al., 2015). Also activity decrease and reduced cortical volumes in the right mid-anterior insula have been associated with impulsivity in non-BPD subjects (Whelan et al., 2012; Churchwell and Yurgelun-Todd, 2013). In comparison, activity in the dorsal ACC responds to behavioral uncertainty, that is, response conflicts (Botvinick et al., 2004). It is proposed to guide attention in manners which limit uncertainty in the information stream (Weissman et al., 2005) and to encode the cost-benefit value of both alternative courses of behavior and the allocation cognitive control (Shenhav et al., 2013; Kolling et al., 2016). The dorsal ACC is thus another prerequisite for optimal attentional and behavioral modulation. In BPD patients, activity aberrance in this area has been associated with impulsivity (Silbersweig et al., 2007; Wingenfeld et al., 2009; Mortensen et al., 2010) as well as emotional dysregulation (Ruocco et al., 2013). In sum, activity aberrance in limbic and prefrontal decision making networks, perhaps the right mid-anterior insula and the dorsal ACC in particular, may contribute to behavioral and emotional dysregulation which is characteristic for patients with BPD (Linehan, 1993). It may also affect attention and cognitive control which can be elucidated in a Posner task.

The Posner task was originally designed to assess an individual’s ability to perform an attentional shift (Posner, 1980). In a typical setup, a directional stimulus (i.e., cue prime), pointing either left or right, is presented at the center of a screen before a target stimulus appears shortly after in either the left or right visual field. Because the cue primes and target stimuli most often correspond in so-called valid trials, the participants are reinforced to direct attention to the primed side. When targets appear in a different location than indicated by the cue primes in invalid trials, the target response times are delayed depending on the participants’ ability to redirect the attention from the primed side. The stronger the cue prime-target correspondence, the more increased becomes the time needed to reorient attention in invalid trials (Yu and Dayan, 2005). Accordingly, attention reorientation can be measured by the reaction time discrepancy between valid and invalid trials. In comparison, impulsive behavior elicited by cue primes can be measured by commission errors in invalid trials, as well as the reaction time discrepancy between valid and neutral trials (i.e., trials with a neutral prime; Avila and Parcet, 2002). Although the paradigm is principally non-emotional, primes provide incomplete information of the upcoming target and thus induce some degree of uncertainty about the upcoming event, i.e., outcome uncertainty (Bach and Dolan, 2012), which can be an important component of distress (Grupe and Nitschke, 2013). Neutral primes yield the highest uncertainty level but elicit no particular expectations. In contrast, cue primes induce both expectations and moderate degrees of uncertainty, which may render distress intolerant individuals, inclined for dichotomous thinking, to overrate the predictive value of the cues. Hence, in addition to attention and cognitive control, the Posner task probes impulsivity, as well as uncertainty processing vs. dichotomous thinking which are relevant aspects in BPD.

The aim of the present study was to elucidate the role of outcome uncertainty and attention-/cognitive control in BPD related impulsivity for the first time, while exploring its underlying neuronal substrates with fMRI at 3T. Uncertainty and associated impulsivity was probed with an event related fMRI adapted modified Posner task. Because neither attention orienting deficits nor its related activity in temporo-parietal brain areas is associated with BPD (Posner et al., 1984, 2002; Doricchi et al., 2010; Krause-Utz et al., 2014), cue primes and targets were presented centrally to minimize the effect of spatial orientation while maximizing the effect of semantic processing. Moreover, the stimulus onset asynchrony between primes and targets was set at 500 ms to allow for conscious processing. These adjustments to the Posner task were made in order to enhance the effect of top-down conscious, dichotomous expectations on impulsive behavior (Avila and Parcet, 2002). Also, the valid/invalid ratio in our design was approximately 3.2:1; yielding a higher degree of outcome uncertainty than a typical Posner task with a ratio of 4:1. The ratio still ensures expectations of prime-target correspondence, but we believed *a priori* that it would enhance the distress related incentive for impulsive responding among BPD patients. We thus hypothesized that cue primes led to impulsive behavior in BPD patients compared to healthy controls (HCs). This would be reflected in more commission errors in invalid trials and quicker responses in valid trials relative to neutral trials. The former measure was of particular interest in the present study as it is a dysfunctional type of impulsivity (Dickman, 1990), and thus more closely related to psychiatric pathology. Furthermore, we expected that brain areas which have previously been shown to be involved in uncertainty processing, attention-/cognitive control and BPD pathology, such as insula, operculum, ACC and middle/superior frontal gyrus (Dosenbach et al., 2006; Paulus and Stein, 2006; Petersen and Posner, 2012; Olsen et al., 2013; Ruocco et al., 2013), would be differentially activated in the BPD patients and HCs during cued vs. neutral prime presentation. In particular, reduced uncertainty processing related to dichotomous and strong expectations during cue primes should be reflected by activity reduction in the right mid-anterior insula in the BPD group.

## Materials and Methods

### Subjects

The study was approved by the regional ethics committee and performed in accordance with the Helsinki Declaration. We invited 15 unmedicated female patients with BPD waiting to be enrolled in an out-patient BPD treatment program at St. Olavs Hospital in Trondheim, Norway. They were diagnosed by certified psychiatrists using standardized diagnostic interviews (SCID I/II). Somatic disease, including neurological disorders/disease, was assessed by evaluation of their medical history. Patients with other psychiatric disorders than BPD were excluded. Other formal exclusion criteria were a history of neurological disorder, head trauma, and MRI contraindications (including claustrophobia) for all participants, as well as psychiatric disorder for HCs. Fifteen female HCs, recruited via flyer advertisement at the hospital/university campus, were matched for age and education. Exclusion criteria were assessed by self-reports. All participants gave written informed consent. One of the HCs was excluded from the data set due to technical errors in the task performance log and one BPD patient was excluded because of a panic attack during the fMRI scan, yielding a final sample of 14 BPD patients and 14 HCs. The mean age of the included participants was 30.1 ± 6.7 for BPD patients and 28.3 ± 7.4 years for HCs (*p* = 0.511), and the mean educational level was 12.1 ± 1.7 years for the BPD patients and 13.1 ± 2 years for HCs (*p* = 0.108). All subjects were right-handed. A self-report measure consisting of impulsivity items from I_7_ (Eysenck et al., 1985) verified that the BPD group was more impulsive than HCs (*t* = 3.678, *p* < 0.001).

### fMRI Task

We adapted a Posner task (Avila and Parcet, 2002) for event-related fMRI compiled in *E*-Prime (Psychology Software Tools, Pittsburgh, PA, USA). Participants were instructed to respond as quickly and accurately as possible by pressing a button with their right or left thumb in response to a defined target stimulus, i.e., a single hatch pointing right (“>”) (right thumb) or a single hatch pointing left (“<”) (left thumb), appearing at the center of the screen. The targets were preceded by centrally placed cue primes, i.e., two small hatches pointing left or right (“<<” or “>>”) or neutral primes, i.e., two small hatches pointing to the center (“><”). A trial was defined as valid if the target was preceded by a corresponding cue prime, invalid if preceded by a discordant cue prime, and neutral if preceded by a neutral prime. Each participant was presented 180 valid, 56 invalid, and 44 neutral trials (a total of 280) in a randomized order (randomize function in *E*-prime) divided into 4 runs. The participants were informed of a predominance of valid trials (but not the exact valid/invalid trial ratio) which ensured expectation of prime-target correspondence.

The task was presented on an LCD screen (Philips Medical Systems, Netherlands) located in the rear of the magnet bore and visible to the participants via a mirror mounted on the head coil. Each prime was displayed for 50 ms followed by a blank screen for 450 ms before the target presentation. The target was displayed for 500 ms, followed by a 2500 or 2600 ms rest period (50/50) plus null-events of different lengths (1800, 3600, 5400, and 7200 ms). Responses were obtained with response grips (Nordic NeuroLab AS, Bergen, Norway) and logged in *E*-Prime. Paradigm presentation and fMRI scanning were synchronized with a SyncBox (Nordic NeuroLab AS, Bergen, Norway). Participants practiced the task outside the scanner until complete task compliance was achieved.

### Behavioral Analyses

Mean reaction times for valid, invalid and neutral trials were calculated after excluding all trials with commission errors, and reaction times < 100 ms. The percentage commission errors were log-transformed to fit the assumption for parametric analyses. Repeated measures analysis of variance (ANOVA) analyses were used to investigate the main effects of trial type on commission errors and reaction times separately, with BPD patients/HCs as a between-subjects factor, followed by paired *t*-tests. Between-group differences were investigated with independent samples *t*-test, and effects sizes (Cohen’s *d*) were calculated. Bonferroni corrected *p*-values (*α* = 0.05) were used to test for significance. Group differences in omission errors were not hypothesized, but were yet analyzed by non-parametric statistics. The results were given as mean (SD) or median (range) depending on data distribution.

### MRI Data Acquisition

MR images were acquired on a Philips Intera 3 Tesla scanner (Philips Medical Systems, Best, Netherlands) with Quasar Dual gradients using a 6-channel sensitivity encoding (SENSE) head-coil (*In Vivo*, Gainesville, USA). The participants’ heads were immobilized using foam padding. During the task, T2*-weighted gradient-echo single-shot echo-planar-imaging (EPI) whole brain measurements were obtained with 42 contiguous axial slices and slice thickness = 4.0 mm, TR = 1800 ms, TE = 35 ms, flip angle = 90°, SENSE reduction factor = 2.2, field-of-view (FOV) = 256, and in plane voxel resolution 2 mm × 2 mm. Four functional runs, each consisting of 182 volumes, were acquired in each participant. Every run was preceded by four dummy scans which were discarded before analysis. A T1-weighted anatomical reference scan was acquired with a 3D MP-RAGE sequence, and a B0 field map was acquired to be used in distortion correction (unwarping).

### MR Image Processing—Whole Brain Analyses

Image analyses were carried out in FSL 4.1.5 (Smith et al., 2004). Motion correction, B0 unwarping, slice timing correction, brain extraction, spatial smoothing (Gaussian kernel FWHM: 5 mm) and high-pass temporal filtering (cut-off: 60 s) were performed. The functional images were registered to the T1W 3D volume and warped to the Montreal Neurological Institute (MNI)-152 standard template using FLIRT (Jenkinson et al., 2002). Statistical analyses were based on FILM, which performs pre-whitening, and fits a general linear model voxel-wise. The expected signal time courses were convolved with a two-gamma hemodynamic response function (Glover, 1999) and its temporal derivative.

To isolate the relevant brain areas to study the effects of uncertain cue primes, the brain activity was modeled with two predictors, cue primes from valid and invalid trials and neutral primes from neutral trials. The predictors started at the prime on-set time and ended at the target on-set time. They formed the contrast cue primes > neutral primes. This contrast isolates the brain activity related to the incomplete information of subsequent targets provided by cues primes, including the effect of the relative uncertainty reduction from neutral primes to cue primes. Also the inverse contrast neutral primes > cue primes was examined in the within group analyses. This contrast isolates the brain activity in the inverse contingency, which includes the relative uncertainty increase in the absence of cues.

Within-subjects parameter estimates were obtained separately for each run, and then pooled across runs with a fixed effects model of variance. Within- and between group analyses were performed with a mixed model of variance. A 4D image for all the subjects was then created by merging these combined parameter estimate images. Non-parametric *t*-tests, using 5000 permutations to build up the null hypothesis, were performed on this image using Randomise with 5 mm variance smoothing (Winkler et al., 2014). Previous studies on BPD related impulsivity have shown that it is difficult to predict under which experimental circumstances BPD patients are impulsive (Sebastian et al., 2013). This also makes it difficult to predict the related brain activity. In addition, the novel view presented here, i.e., a possible role outcome uncertainty in distress related impulsivity, investigated using an event related paradigm led us to threshold the *t*-statistic images with liberal uncorrected voxel *p*-values < 0.005 (*t* = 2.78) and a cluster size threshold of ≥20 voxels. This threshold strategy has previously been recommended by Lieberman and Cunningham (2009) for explorative purposes as it reduces the risk for type 2 errors, with the expense of type 1 errors. Their simulations also showed that it corresponded to FDR correction of *q* < 0.05 under common imaging parameters. A similar thresholding strategy was recently found to provide solid data reproducibility compared to both FDR and familywise error rate correction (Roels et al., 2016). Effect sizes (Cohen’s *d*) were estimated in the hypothesized brain areas based on the mean *Z*-values for each participant in the significant clusters.

## Results

### Task Performance

The repeated measures ANOVA showed main effects of trial type on commission errors (*F*_(2,52)_ = 41.473, *p* < 0.001) and reaction times (*F*_(2,52)_ = 138.051, *p* < 0.001). There were more commission errors in invalid trials vs. neutral trials (*t =* 5.658, *p* < 0.001), and more in neutral trials than valid trials (*t* = 2.174, *p* = 0.039). Mean reaction times in valid trials were significantly shorter than in neutral trials (*t =* −14.013, *p* < 0.001). Mean reaction times in neutral trials were shorter than in invalid trials (*t* = −3.387, *p* = 0.002). There were interaction effects between trial type and group (BPD vs. HCs) for both reaction times (*F*_(2,52)_ = 7.772, *p* = 0.003) and commission errors (*F*_(2,52)_ = 12.926, *p* < 0.001). The interaction effects entailed that BPD patients, compared to HCs, went from quicker towards slower responses and more commission errors across trials, from valid to neutral to invalid trials.

Task performance and between-group differences are displayed in Table 1. BPD patients made significantly more commission errors than HCs in invalid trials. Although there were no significant trial-trial reaction time differences between groups, BPD patients had a significantly larger difference between reaction times in neutral trials and valid trials than HCs. This demonstrates a greater impact of cue priming among BPD patients on reaction times. All participants made few omission errors with a median (range) of 0.4 (0–4.6)%, and no significant group differences were found.

**Table 1:** **Performance on modified Posner task in borderline personality disorder (BPD) and healthy control (HC) participants**.

Variable	BPD	HC	*p*-value	Cohen’s *d*	*t*-score
*Commission errors (%), median (range)*
**Invalid trials**	**20 (1.8–37.5)**	**3.6 (0–17.9)**	**0.007^a^**	**1.413**	**3.602**
Neutral trials	4.5 (0–34.1)	0 (0–9.5)	0.098	0.935	2.383
Valid trials	1.7 (0–7.1)	1.7 (0–4.5)	1	0.175	0.445
*Reaction times (ms), mean (SD)*
Invalid trials	475.7 (40.4)	460.0 (25.0)	1	0.227	1.237
Neutral trials	466.1 (37.9)	454.6 (28.7)	1	0.18	0.904
Valid trials	395.8 (37.1)	412.0 (26.3)	1	−0.503	−1.339
**Neutral—Valid**	**70.3 (19.4)**	**42.6 (21.2)**	**0.007^a^**	**1.363**	**3.612**

*The percentage commission errors were log-transformed to fit the assumption for parametric analyses. Between-group differences were investigated with independent samples t-test, and effects sizes (Cohen’s d) were calculated. Bonferroni corrected p-values (α = 0.05) were used to test for significance. ^a^Corrected p-value below the significance threshold*.

### fMRI Data—Whole Brain Analyses

The within-group analyses of brain activity for the contrast cue primes > neutral primes in BPD patients demonstrated significant activity in the left dorsal ACC, cerebellum, superior frontal gyrus, as well as bilaterally in the paracingulate and middle frontal gyrus (Table 2, Supplementary Figure S1). For neutral primes > cue primes, the BPD group had significant activity in the right rostrolateral prefrontal cortex, postcentral and temporal gyrus, as well as the left temporal and occipital pole and bilaterally in cerebellum (Table 2, Supplementary Figure S1). The HC group had significant activity in the right superior temporal gyrus, cerebellum, pregenual ACC, temporal pole, the left superior/middle frontal, postcentral, precentral and lingual gyrus, temporal fusiform, lateral orbitofrontal and occipital cortex, as well as bilaterally in the middle temporal gyrus in the cue primes > neutral primes contrast (Supplementary Table S1, Supplementary Figure S2). In the neutral prime > cue prime contrast, HCs had significant activity in the right precentral and superior frontal gyrus, rostrolateral prefrontal cortex, cerebellum, the left temporoparietal junction, as well as bilaterally in the frontal operculum (Supplementary Table S2, Supplementary Figure S2).

**Table 2:** **Significant within-group activations in the cue primes > neutral primes and neutral primes > cue primes contrast among borderline personality disorder patients**.

Anatomical region (left/right)	Cluster size	Max *t*-scores MNI coordinates: *x, y, z*	Max *t*-scores
**Cue primes > Neutral primes**
Anterior cingulate cortex (L)	96	−10, 44, 14	4.49
Cerebellum (L)	21	−12, −38, −16	3.94
Middle/superior frontal gyrus (L)	101	−30, 28, 54	3.78
Paracingulate gyrus (L/R)	34	0, 28, 38	3.75
Middle frontal gyrus (R)	31	42, 32, 40	3.39
**Neutral primes > Cue primes**
Rostrolateral prefrontal cortex (R)	55	28, 54, 4	4.23
Postcentral gyrus (R)	35	62, −18, 46	4.08
Middle temporal gyrus (R)	21	64, −52, 12	3.97
Temporal pole (L)	51	−60, 8, −2	3.96
Occipital pole (L)	98	−34, −92, −18	3.73
Cerebellum (L/R)	42	−32, −54, −26	3.53
	34	34, −58, −24	3.52

*Whole-brain, non-parametric permutation analysis with a statistical threshold of uncorrected p < 0.005, cluster size ≥ 20 corresponding to a FDR-correction of q < 0.05; R, right, L, left*.

**Figure 1:**
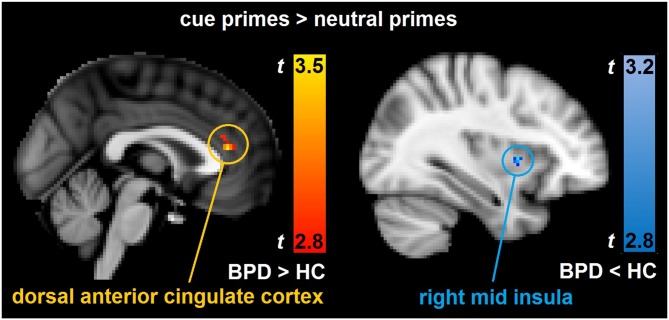
**Depicts the significant between-group brain activity differences in the hypothesized brain areas.** The results are derived from whole-brain, non-parametric permutation analyses with a statistical threshold of uncorrected *p* < 0.005, cluster size ≥ 20 corresponding to a FDR-correction of *q* < 0.05. Compared to healthy controls (HC), borderline personality disorder (BPD) showed increased brain activity bilaterally in the dorsal anterior cingulate cortex (ACC; peak voxel Montreal Neurological Institute (MNI) coordinates: *x* = 0, *y* = 36, *z* = 14) and decreased brain activity in the right mid insula (peak voxel MNI coordinates: *x* = 34, *y* = 6, *z* = 4) in the contrast cue primes > neutral primes.

In the between group analyses of cue primes > neutral primes, the BPD patients had significantly increased brain activity relative to HCs bilaterally in the dorsal ACC (Table 3, Figure 1). In the same contrast, BPD patients had significantly reduced brain activity compared to HCs in the right mid insula (Table 3, Figure 1), rostrolateral prefrontal cortex, temporal pole, superior/middle temporal and precentral gyrus, the left cerebellum, putamen, postcentral gyrus, temporal fusiform and lateral occipital cortex, as well as bilaterally in the ventromedial prefrontal cortex (Table 3). Thus, activity aberrance in BPD patients was revealed in hypothesized brain areas during uncertain cue primes, that is, activity increase in the dorsal ACC and activity decrease in the right mid insula, and the measured effect sizes were large, *d* = 1.24 and −1.63 respectively.

**Table 3:** **Significant between-group activations in the cue-primes > neutral primes contrast**.

Anatomical region (left/right)	Cluster size	Max *t*-scores MNI coordinates: *x, y, z*	Max *t*-scores
**BPD > HC**
Anterior cingulate cortex (L/R)	22	0, 36, 14	3.47
**HC > BPD**
Postcentral gyrus (L)	44	−66, −6, 20	4.49
Temporal pole (R)	57	58, 6, −28	4.43
	80	62, 10, −6	3.73
Superior temporal gyrus (R)	32	50, 2, −6	3.84
Premotor cortex (R)	25	12, −28, 50	3.82
Cerebellum (L)	83	−16, −64, −28	3.77
Temporal fusiform cortex (L)	60	−30, −44, −22	3.74
Rostrolateral prefrontal cortex (R)	29	28, 54, 8	3.63
Lateral occipital cortex (L)	33	−28, −92, 32	3.58
Ventromedial prefrontal cortex (L/R)	39	−4, 40, −18	3.34
Putamen (L)	24	−28, 0, 8	3.21
Mid insula (R)	21	34, 6, 4	3.16
Middle temporal gyrus (R)	21	50, −34, −4	3.12

*Whole-brain, non-parametric permutation analysis with a statistical threshold of uncorrected p < 0.005, cluster size ≥ 20 corresponding to a FDR-correction of q < 0.05; BPD = borderline personality disorder; HC = healthy controls; R, right, L, left*.

## Discussion

The present study investigated the brain activity and the dysfunctional impulsive behavior associated with how outcome uncertainty led BPD patients to overrate the predictive value of cue primes. In line with our hypotheses, BPD patients, in comparison to HCs, showed more dysfunctional impulsive behavior as measured by increased commission errors in invalid trials, that is, to incorrectly primed targets. Furthermore, BPD patients showed a significantly larger reaction time decrement, from neutral to valid trials, which supports a stronger impact of cue primes in BPD patients than in the HC group. Also in line with our hypotheses, the between group analyses revealed that BPD patients had a larger brain activity reduction than HCs in the right mid insula during cue primes, which is interpreted as a reduction of distress and outcome uncertainty due to strong and dichotomous expectations for the upcoming event. In addition, BPD patients had a larger brain activity increase than HCs bilaterally in the dorsal ACC during cue primes, which may indicate a strong readiness to respond.

In the present Posner task, uncertain cue primes induced dysfunctional impulsive behavior, measured by commission errors in BPD patients, in accordance with the current BPD population’s self-reported personality profiles (i.e., I_7_) and core elements of their diagnosis (American Psychiatric Association, 2013). Because the Posner task induced outcome uncertainty, an important component in distress (Grupe and Nitschke, 2013), the impulsive behavior may arise from distress intolerance. Distress (or uncertainty) intolerance may render the BPD patients to overrate the predictive value of environmental cues in order to reduce distress. This tendency may be a feature of dichotomous thinking (Kernberg, 1975). Without cues, high levels of uncertainty may induce chaotic behavior, e.g., restrictive *and* impulsive behavior, as indicated in the present study by the (non-significant) tendencies in BPD patients for slower responses and more commission errors in neutral trials (Table 1). Because the Posner task is otherwise non-emotional, the implication of this finding is that BPD patients may be particularly sensitive to uncertainty components in distress.

Previous studies on BPD and distress related impulsivity have shown that BPD participants behave predominantly impulsive, restrictive, or both. In one study, subjects with high levels of BPD related traits were found to be predominantly more impulsive (i.e., commission errors) in a passive avoidance learning task after having watched a thriller movie chase scene (Chapman et al., 2010) which induces tension, fear and interest (Gross and Levenson, 1995), and intuitively outcome uncertainty. Distress imposed by concomitant perceptual noise, aversive pictures and time pressure were also found to increase impulsivity (i.e., commission errors; Cackowski et al., 2014). Here, stimulus overload should yield high levels of perceptual noise and thus sensory uncertainty (Bach and Dolan, 2012). During the stimulation of negative affective words, BPD patients were found to be both more impulsive and restrictive (i.e., increase in commission errors and slow responses), compared to HCs, in Stroop tasks (Arntz et al., 2000; Wingenfeld et al., 2009), and in a go/no-go task (i.e., commission and omission errors; Silbersweig et al., 2007). In comparison, no group differences were found between BPD patients and HCs in a go/no-go task after anger induction, specifically (Jacob et al., 2013). Finally, restrictive behavior (i.e., reduced commission errors) has been reported in a passive avoidance learning task where negative emotional states were present at baseline in participants with BPD related traits (Chapman et al., 2008). These mixed results may reflect BPD patients’ impulsive and avoidant personality profiles (Saulsman and Page, 2004) and that impulsivity is only one aspect of an overall extreme behavioral response pattern. Moreover, in addition to the present study, the two other studies which found predominantly impulsive behavior in BPD patients included manipulations where outcome and sensory uncertainty were important components (Chapman et al., 2010; Cackowski et al., 2014). These uncertainty types are previously shown to influence behavior in manners which seek to limit uncertainty (Bach and Dolan, 2012) and may accordingly be prerequisites for dichotomous thinking and dysfunctional impulsivity in BPD.

The results of the present study supported our hypotheses, i.e., that those brain areas which are previously shown to be involved in uncertainty processing, attention/cognitive control and BPD pathology should be aberrant among BPD patients in the present task. Specifically, the analysis of brain activity related to the contrast cue primes > neutral primes revealed that the BPD patients had a greater reduction of brain activity in the right mid insula. Review articles, in general, ascribe a particular role for the right *anterior* insula in distress and uncertainty processing (Paulus and Stein, 2006; Bach and Dolan, 2012; Grupe and Nitschke, 2013). But also the right *mid* anterior insula has been found to be active during conditions of uncertainty (Paulus and Frank, 2006; Simmons et al., 2008; Sarinopoulos et al., 2010; Becker et al., 2014) and related to both distress and response inhibition during distress in BPD (Silbersweig et al., 2007; Dziobek et al., 2011). In comparison to the anterior insula, which provides the highest level of information integration (Craig, 2009) and is primarily connected to prefrontal brain regions, the mid insula connects both with prefrontal cognitive-emotional and higher-order sensory regions (Wiech et al., 2014). Accordingly, it has previously been suggested that the mid insula integrates cognitive-emotional and highly processed sensory information (Craig, 2009), and possibly, outcome uncertainty related to primes in the present study. Importantly, the present results reflect the *relative* difference in uncertainty processing between neutral primes and cue primes, in which the former condition perhaps induced higher subjective levels of outcome uncertainty in BPD patients, compared to HCs, reflected by a right mid insular activity increase. Yet, the strong impact of cue primes on commission errors suggests that the relative activity difference also reflects that the BPD patients overrated the cues’ predictive value, which led to reduced outcome uncertainty, and ultimately, dysfunctional impulsive behavior.

Another prediction was confirmed in the contrast cue primes > neutral primes, where the BPD patients had increased activity bilaterally in the dorsal ACC compared to HCs. This area of the dorsal ACC is proposed to encode the value of an alternative course of behavior than the current and to invigorate new responses (Kolling et al., 2016). Thus, the increased activity in the present task may reflect an inclination to respond (i.e., the alternative behavior) rather than to wait (i.e., the current behavior) and delay the response engagement until the target is evident in the BPD group. Meanwhile, the suppression of an already initiated response is proposed to depend on the subthalamic nucleus, a part of the basal ganglia (Mink, 1996; Aron and Poldrack, 2006). This area has previously been found hyperactive in BPD patients during response inhibition in a go/no-go task after anger induction, but not in a neutral condition (Jacob et al., 2013). It indicates that BPD patients need to engage a strong inhibitory effort during distress, in order to neutralize the increased inclination to act demonstrated in the present study.

In sum, the results of the present study indicate that aberrant brain activity in the mid right insula and dorsal ACC in BPD patients are of particular importance for BPD related impulsivity. Moreover, we suggest that the activity patterns in the right mid insular and dorsal ACC reflected distress (or uncertainty) intolerance in BPD patients. Interestingly, a self-report measure of uncertainty intolerance has been associated with activity aberrance in both right mid insula (Simmons et al., 2008) and the dorsal ACC in non-BPD subjects (Krain et al., 2006, 2008) providing further support to the notion of a particular role of the right mid insula and the dorsal ACC for uncertainty and distress management based on the current results.

The present study has several limitations. First, the study population was small, but the inclusion of unmedicated, female out-patients with BPD without significant comorbidity was a strength with regard to the homogeneity of the study population. Without co-morbidities, the results of the present study could be particularly specific for BPD. Also by including unmedicated patients, unwanted effects of pharmacological substances on the fMRI signal were avoided. Moreover, the group differences detected should represent a minimum as the most severely affected patients were not eligible for inclusion. A small study sample does, however, increase the risk of both type 1 and 2 mistakes (i.e., false negative results while true effects may be exaggerated; Button et al., 2013). Secondly, the study design included a sub-optimal jitter for the stimulus onset interval relative to TR, which may have increased the risk of false negative results further (Huettel et al., 2004). Thirdly, the *a priori* statistical threshold implemented in the fMRI analyses recommended by Lieberman and Cunningham (2009), allows for more subtle effects to become significant but at the expense of an increased risk for false positives. Some compensation for these shortcomings was provided by the non-parametrical analysis and permutation testing which increases the power and reduces the risk for exaggerated effects provided by statistical outliers (Winkler et al., 2014). In addition, the effect sizes of the hypothesized behavioral and brain activity differences were found to be large. In sum, the results must be viewed as preliminary and need replication.

In conclusion, the significant differences in commission errors, reaction times, and brain activity between the BPD and HC groups demonstrate that BPD patients overrated the predictive outcome value of uncertain cue primes. The strong expectations led to reduced uncertainty, increased response readiness, and ultimately, dysfunctional impulsivity in BPD patients. These tendencies may be aspects of dichotomous thinking and distress intolerance. In addition, outcome uncertainty may be an important component in distress related impulsivity in BPD.

## Author Contributions

JAM and AKH have been involved in all parts of the process, planning, recruiting study subjects, execution of the experiment, data analyzing and writing the manuscript. HRE has contributed in data analyzing and writing the manuscript. GK has contributed in the planning phase, recruiting study subjects and writing the manuscript.

## Funding

The study was funded by the Faculty of Medicine, the Norwegian University of Science and Technology (NTNU) and Tiller Psychiatric Center, St. Olavs Hospital.

## Conflict of Interest Statement

The authors declare that the research was conducted in the absence of any commercial or financial relationships that could be construed as a potential conflict of interest.
