# Supplementary material for: Outcome Uncertainty and Brain Activity Aberrance in the Insula and Anterior Cingulate Cortex Are Associated with Dysfunctional Impulsivity in Borderline Personality Disorder
Source: Front Hum Neurosci. 2016 May 6;10:207. doi: 10.3389/fnhum.2016.00207 (PMC4858533; doi:10.3389/fnhum.2016.00207)
Supplement: Supplementary file 1 [file DataSheet_1.docx]

**Supplementary material**

**Table S1.** *Significant within-group activations in the cue-primes > neutral primes contrast among healthy controls*

| Anatomical region (left/ right) | Cluster size | Max t-scores MNI  coordinates: x, y, z | Max t-scores |
| --- | --- | --- | --- |
| Rostromedial prefrontal cortex (R) | 69 | 10, 68, 30 | 4.98 |
| Middle temporal gyrus (L/ R) | 201 | -60, 0, -28 | 4.86 |
|  | 105 | 60, 2, -32 | 4.47 |
| Cerebellum (R) | 137 | 18, -28, -24 | 4.62 |
| Middle frontal gyrus (L) | 63 | -32, 22, 58 | 4.42 |
| Temporal fusiform cortex (L) | 81 | -32, -42, -18 | 4.33 |
| Postcentral gyrus (L) | 32 | -66, -6, 22 | 4.08 |
| Superior frontal gyrus (L) | 20 | -14, 40, 54 | 4.07 |
| Putamen (L) | 31 | -24, -4, 14 | 3.97 |
| Superior temporal gyrus (R) | 23 | 46, -16, -2 | 3.92 |
| Anterior cingulate cortex (R) | 161 | 4, 40, 0 | 3.79 |
| Lateral orbitofrontal cortex (L) | 34 | -34, 20, -22 | 3.72 |
|  | 43 | -48, 32, -8 | 3.5 |
| Lingual gyrus (L) | 98 | -12, -60, 4 | 3.57 |
| Precuneus (L) | 30 | -2, -58, 22 | 3.7 |
|  | 30 | -18, -62, 16 | 3.46 |
| Temporal pole (R) | 77 | 62, 10, -4 | 3.4 |
| Precentral gyrus (L) | 29 | -46, -14, 60 | 3.29 |
| Lateral occipital cortex (L) | 30 | -38, -82, 40 | 3.25 |

*Whole-brain, non-parametric permutation analysis with a statistical threshold of uncorrected p < 0.005, cluster size ≥ 20 corresponding to a FDR-correction of q < 0.05; R, right, L, left*

**Table S2.** *Significant within-group activations in the neutral primes > cue primes contrast among healthy controls*

| Anatomical region (left/ right) | Cluster size | Max t-scores MNI  coordinates: x, y, z | Max t-scores |
| --- | --- | --- | --- |
| Temporoparietal junction (L) | 44 | -66, -36, 28 | 3.88 |
| Precentral gyrus (R) | 21 | 54, 8, 44 | 3.88 |
|  | 27 | 36, -20, 50 | 3.4 |
| Frontal operculum (L/ R) | 38 | -34, 28, 4 | 3.81 |
|  | 32 | 34, 26, 6 | 3.52 |
| Rostrolateral prefrontal cortex (R) | 45 | 48, 52, 10 | 3.64 |
| Cerebellum (R) | 29 | 42, -84, -30 | 3.57 |
| Superior frontal gyrus (R) | 26 | -20, -10, 76 | 3.48 |

*Whole-brain, non-parametric permutation analysis with a statistical threshold of uncorrected p < 0.005, cluster size ≥ 20 corresponding to a FDR-correction of q < 0.05; R, right, L, left*

**Figure S1**

**
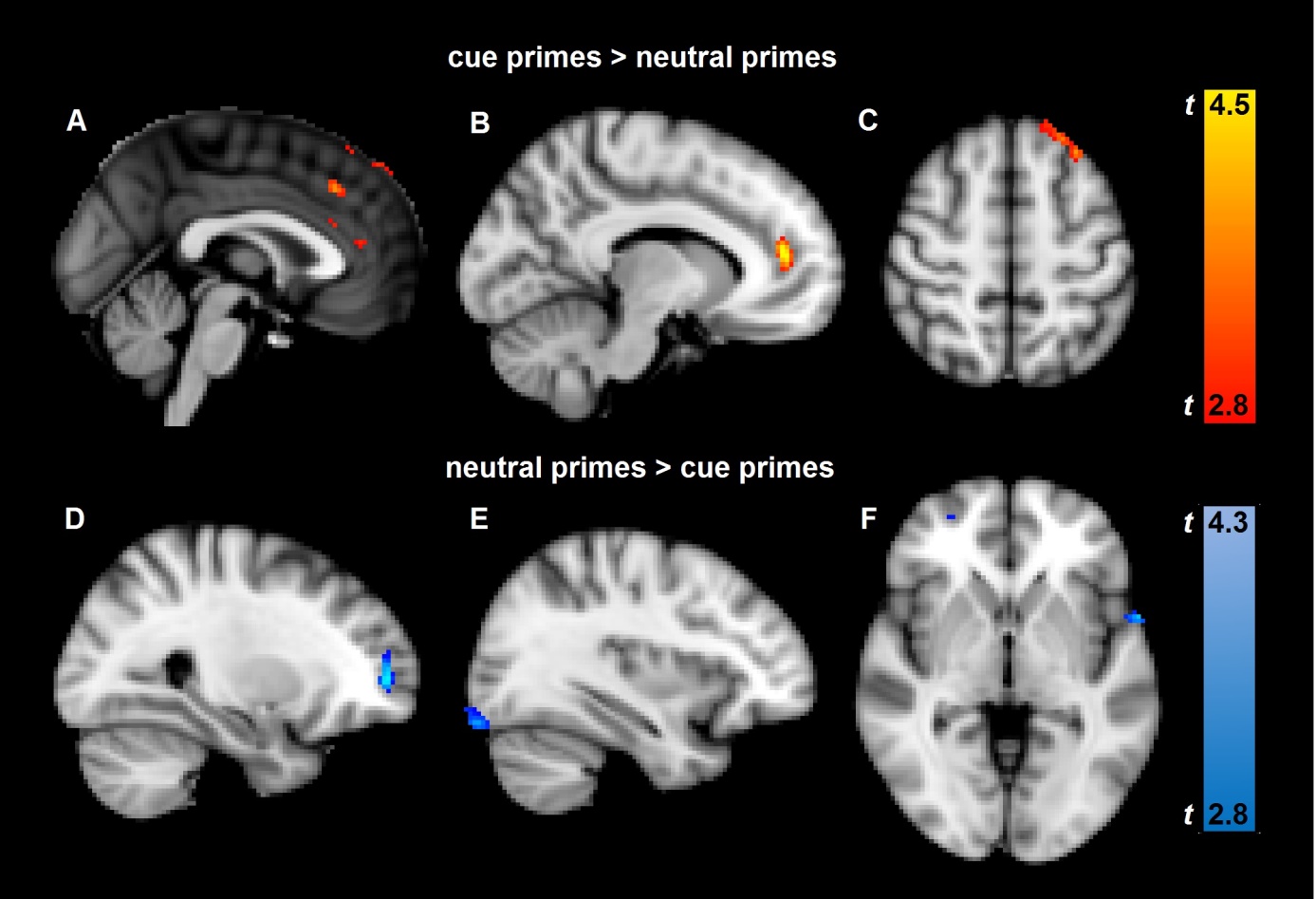
**

*Figure S1 depicts significant within-group brain activity in the group of patients with borderline personality disorder. The results are derived from whole-brain, non-parametric permutation analyses with a statistical threshold of uncorrected p < 0.005, cluster size ≥ 20 corresponding to a FDR-correction of q < 0.05. A (sagittal plane, x = 0), B (sagittal plane, x = -10) and C (transverse plane, z = 54) depicts brain activity from the contrast cue primes > neutral primes in the left paracingulate gyrus (A), left anterior cingulate cortex (A, B) and the left middle-/ superior frontal cortex (A, C). D (sagittal plane, x = 28), E (sagittal plane, x = -34) and F (transverse plane, z = -2) depicts brain activity from the contrast neutral primes > cue primes in the right rostrolateral prefrontal cortex (A, C), the left occipital pole (B) and the left temporal pole (C).*

**Figure S2**

**
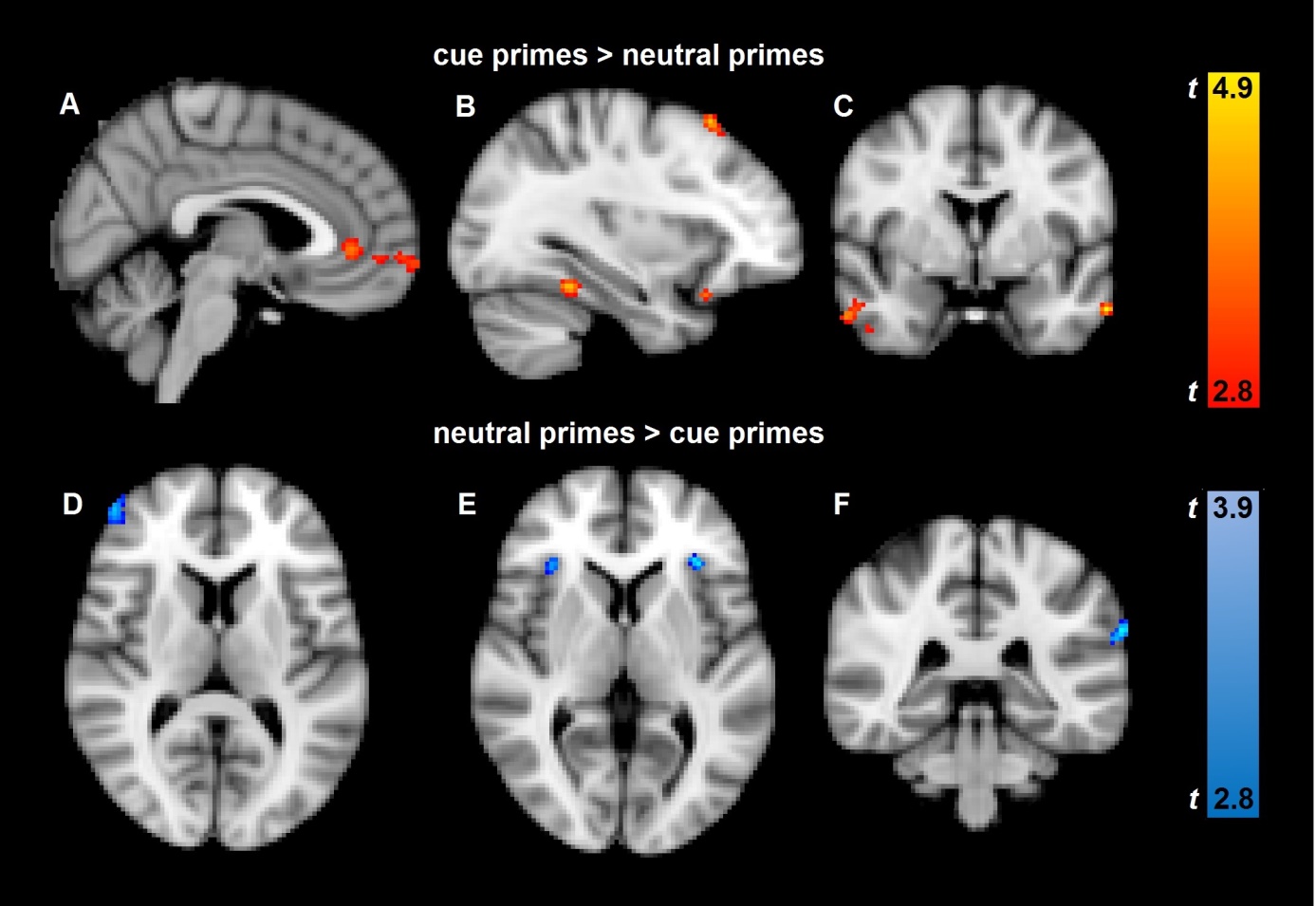
**

*Figure S2 depicts significant within-group brain activity in the group of healthy controls. The results are derived from whole-brain, non-parametric permutation analyses with a statistical threshold of uncorrected p < 0.005, cluster size ≥ 20 corresponding to a FDR-correction of q < 0.05. A (sagittal plane, x = 4), B (sagittal plane, x = -32) and C (coronal plane, y = 0) depicts brain activity from the contrast cue primes > neutral primes in the right anterior cingulate and rostromedial cortex (A), left middle frontal gyrus, temporal fusiform and lateral orbitofrontal cortex (B) and bilaterally in the middle temporal gyrus (C). D (transverse plane, z = 10), E (transverse plane, z = 4) and F (coronal plane, y = -36) depicts brain activity from the contrast neutral primes > cue primes in the right rostrolateral prefrontal cortex (A), bilaterally in the frontal operculum (B) and the left temporoparietal junction (C).*
